# A Case of Epilepsy in a Boy, Successfully Treated

**Published:** 1846-07

**Authors:** Thomas C. Osborne

**Affiliations:** Erie, Alabama


### Art. II.—*A Case of Epilepsy in a Boy, successfully treated.* By Dr. Thomas C. Osborne, of Erie, Alabama.

Servitus, a boy 5 years of age, well grown, and sprightly, was attacked with epilepsy in 1843, without any apparent cause. For more than two years the disease continued to recur periodically, and, in most instances, at the time of the full moon, though a few times at other lunar periods. Always for some days before an attack his bowels were observed to become costive, the stools being scanty, hard, and clay-colored. The paroxysms commenced before he awoke in the morning, or immediately after he rose from bed, and continued to recur regularly every two hours until night, attended by exhaustion, and moroseness of temper. The fits lasted about one minute, and were followed by a profound sleep for half an hour, from which he uniformly awoke complaining of pain in his head. In a short time another followed, but in the intervals, he was conscious of what had occurred, and had a presentiment of what was approaching.

The phenomena frequently observed in one of the paroxysms were, lividness of the lips, pallor of the face, copious secretion of saliva, a vacant and fatuous stare, slight tremors, accelerated pulse, suspended respiration, and convulsive efforts to swallow. If sitting in a chair when a fit came on, he leaned forward, pointing his finger, as if coursing some object around the room, the agitation of his body and convulsive action of his throat being greatly increased by the erect posture. Once while thus sitting his mother spoke sharply to him, at the same time touching him on the shoulder, whereupon he started up, looked around him, drew a long breath, and complained of feeling sleepy; the fit was dispelled.

The boy had been treated by two physicians before I saw him; the first treated the case as epilepsy dependent on worms; the second regarded the liver as being in fault, and shaped the treatment accordingly. It was in April, 1845, that the case was placed under my direction. I inserted a seton in the nape of the patient's neck; and prescribed the following pill:—nitrate of silver one-fifth of a grain, extract of gentian two grains; one to be taken three times a day.

May 12th. The patient has experienced a paroxysm of the disease. I ordered the nitrate of silver to be increased to $\frac{1}{4}$th of a grain in the pill, and the pills to be continued. The magneto-electrical machine was applied, one wire to the nape of the neck, and the other carried successively over the front of the neck, pit of the stomach, and right hypochondrium. This application was directed to be made two days preceding each change of the moon; and calomel in doses of two grains, was given morning and evening for three days previous to each such period.

June 12th. The lunar period has passed, and the boy has not been affected by one of his paroxysms. The same treatment to be continued.

July 12th. No return of the disease. Continued the treatment.

August 12th. As he remained free from all symptoms of disease, the treatment was suspended; but in September he had a recurrence of the malady, and the use of the pills and electricity was resumed.

Nov. 12th. To-day he was seized, contrary to the previous progress of his disease, between the full and last quarter of the moon. I now ordered a renewal of the seton, and that the pills should be given for a longer time before the expected paroxysm, the amount of nitrate of silver in each to be increased to one-third of a grain.

From that time to the present, a period of nearly six months, he has remained free from the complaint.

May 8th, 1846.

